# Quasi-bound states in an NPN-type nanometer-scale graphene quantum dot under a magnetic field

**DOI:** 10.1038/s41598-020-77357-8

**Published:** 2020-11-24

**Authors:** Yueting Pan, Haijiao Ji, Xin-Qi Li, Haiwen Liu

**Affiliations:** 1 Center for Advanced Quantum Studies, Department of Physics, Beijing Normal University, Beijing, 100875 China; 2 Center for Joint Quantum Studies, School of Science, Tianjin University, Tianjin, 300072 China; 3 Department of Physics, School of Science, Tianjin University, Tianjin, 300072 China

**Keywords:** Condensed-matter physics, Theory and computation, Condensed-matter physics

## Abstract

We solve the quasi-bound state-energy spectra and wavefunctions of an NPN-type graphene quantum dot under a perpendicular magnetic field. The evolution of the quasi-bound state spectra under the magnetic field is investigated using a Wentzel–Kramers–Brillouin approximation. In numerical calculations, we also show that the twofold energy degeneracy of the opposite angular momenta breaks under a weak magnetic field. As the magnetic field strengthens, this phenomenon produces an observable splitting of the energy spectrum. Our results demonstrate the relation between the quasi-bound state-energy spectrum in graphene quantum dots and magnetic field strength, which is relevant to recent measurements in scanning tunneling microscopy.

## Introduction

Graphene has promising prospects in condensed matter physics owing to its extraordinary properties, such as Klein tunneling, Zitterbewegung motion, and minimum quantum conductance^1–4^. These properties allow the investigation and realization of exotic relativistic quantum phenomena^3^. Although Klein tunneling precludes the capture of relativistic quasi-particles in graphene, the energy barrier in graphene nano-structures such as PN junctions can induce quasi-bound states with finite lifetimes^5^. Quasi-bound states in graphene quantum dots and other graphene constrained systems have been prominently studied in theoretical works^5–9^, and experiments^10–14^. For example, the found quasi-bound states are related to the atomic collapse states that have been found for the Coulomb problem^15–17^.

The quantum Hall effect in graphene systems under a magnetic field has been widely studied recently. Meanwhile, in graphene quantum dots under a magnetic field, the quasi-bound states coexist with the magnetic field, resulting in interesting physical phenomena. Thus far, graphene quantum dots under magnetic fields have been studied in previous works^18–22^, however qualitative semi-classical and numerical analyses can help us to interpret the energy spectra and physical properties of graphene quantum dots under magnetic fields more completely.

In this paper, we study the wavefunction, local density of states (LDOS) and energy spectrum of an NPN-type circular graphene quantum dot under a magnetic field. The numerical results are based on the Wentzel–Kramers–Brillouin (WKB) approximation. Comparing the zero-field results of the WKB approximation with rigorous analytical results, we find energy degeneracy in the opposite angular momenta. Next, the energy spectra under different magnetic fields are obtained by the WKB approximation. Increasing the magnetic field breaks the energy degeneracy of the opposite angular momenta, leading to increasingly obvious splitting in the spectrum. This LDOS splitting is also observable in experiments.

**Figure 1:**
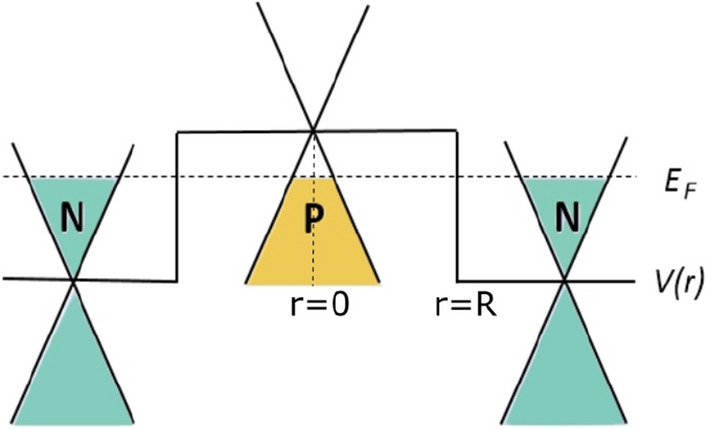
Energy band of an NPN-type graphene quantum dot with Fermi energy $E_{F}$ and potential *V*(*r*). Quasi-bound states are formed in section ${\mathrm {P}}$, and the substrate is section ${\mathrm {N}}$, where the *V*(*r*) is higher in section ${\mathrm {P}}$ than in section ${\mathrm {N}}$.

The remainder of this paper is organized as follows. First, we model a graphene quantum dot under a magnetic field by the Dirac equation and numerically solve the wavefunction and quasi-bound states spectrum by the WKB approximation. The analytical solution without a magnetic field is also presented. After evolving the energy spectrum under a magnetic field, we conclude the study and highlight its relevance to recent experiments.

## Modeling and radial function

We consider an NPN-type graphene quantum dot in a perpendicularly upward homogeneous magnetic field. The Dirac equation of this system is given by^23–25^:

1 $$\begin{aligned}&\left( i\hbar \partial _{t}-\hat{H}\right) \psi (r,t)=0,\nonumber \\&\quad \hat{H}=\sigma _{\mu }P_{\mu }+V(r)=\sigma _{\mu }\left( -i\hbar \partial _{\mu }+eA_{\mu }\right) +V(r),\,\mu =x,y, \end{aligned}$$

where the Fermi velocity is set to $v_{F} = 1$, and $\sigma _{\mu }$ is the Pauli matrices. Fig. 1^5,6,8,11^ shows the centrally symmetric step potential *V*(*r*) generated in the NPN-type circular graphene quantum dot:

2 $$\begin{aligned} V(r)=\Big\{ \begin{array}{lcr} V_{0}, &{}r<R\\ 0, &{}r>R. \end{array} \end{aligned}$$

where *R* is the radius of the graphene quantum dot, $P_{\mu }$ is the generalized momentum, and $A_{\mu }$ is the vector potential generated by the perpendicular magnetic field *B* along the z-axis, calculated as $A_{x}=-\frac{By}{2},A_{y}=\frac{Bx}{2}$.

The wavefunction is assumed as $\psi (r,\varphi ,t)=\frac{1}{\sqrt{2\pi }}e^{-iEt/\hbar }\psi _{l}(r,\varphi ),$ where $E=E_{F}-E_{D}$ is the difference between the Fermi energy $E_{F}$ and the Dirac-point energy $E_{D}$. The eigenfunction of the total angular momentum *l* takes the form:

3 $$\begin{aligned} \psi _{l}(r,\varphi )=\frac{1}{\sqrt{r}} \left( \begin{array}{lcr} F(r)e^{il\varphi }\\ G(r)e^{i(l+1)\varphi } \end{array} \right) \end{aligned}$$

where *l* is the angular momentum quantum number. Further, using $P_{x}\pm iP_{y}=-ie^{\pm i\varphi }[\hbar \partial _{r}\pm (\frac{i\hbar }{r}\partial _{\varphi }-\frac{eBr}{2})],$ the equation can be recast as

4 $$\begin{aligned}&\hbar \frac{{\mathrm {d}}F}{{\mathrm {d}}r}-\left( \frac{\left( l+\frac{1}{2}\right) \hbar }{r}+\frac{eBr}{2}\right) F+\left( E-V(r)\right) G=0; \end{aligned}$$

5 $$\begin{aligned}&\hbar \frac{{\mathrm {d}}G}{{\mathrm {d}}r}+\left( \frac{\left( l+\frac{1}{2}\right) \hbar }{r}+\frac{eBr}{2}\right) G-\left( E-V(r)\right) F=0. \end{aligned}$$

In the next section, we solve the coupled differential equations in Eqs. (4) and (5) using the WKB approximation.

## WKB solutions in the presence and absence of a magnetic field

### WKB approximation

In the absence of a magnetic field, the energy spectra of a disc can be accurately described by the Bessel function^11^; however, finding the rigorous solution of a graphene quantum dot under a magnetic field is considerably difficult. Thus, we apply the WKB approximation^26^ to a quantum dot under a magnetic field. To obtain the WKB form, we substitute $(l+\frac{1}{2})\hbar$ in Eqs. (4) and (5) by $m\equiv l+\frac{1}{2} =\pm \frac{1}{2},\pm \frac{3}{2},\pm \frac{5}{2}\cdots$ and retain the $\hbar$ in front of the differential sign as the expansion parameter^27^. In matrix form, this becomes

6 $$\begin{aligned} \hbar \left( \begin{array}{lcr} F'(r) \\ G'(r) \end{array} \right) =D\left( \begin{array}{lcr} F(r) \\ G(r) \end{array} \right) , \end{aligned}$$

where

7 $$\begin{aligned} D=- \left( \begin{array}{lcr} -(\frac{m}{r}+\frac{eBr}{2}) &{} E-V(r) \\ -E+V(r) &{} \frac{m}{r}+\frac{eBr}{2} \end{array} \right) . \end{aligned}$$

We then suppose

8 $$\begin{aligned} F(r)=\beta (r)e^{i\frac{y(r)}{\hbar }};G(r)=e^{i\frac{y(r)}{\hbar }}, \end{aligned}$$

in which $\beta (r)$ is the difference factor between the upper- and lower-component wavefunctions, and *y*(*r*) is the local phase^28^. Writing *y*(*r*) as a Taylor expansion

9 $$\begin{aligned} y(r)=\sum _{n=0}^{\infty }\hbar ^{n}y_{n}(r), \end{aligned}$$

the solution reads

10 $$\begin{aligned} y'_{0}(r)= \pm \sqrt{(E-V(r))^{2}-\left(\frac{m}{r}+\frac{eBr}{2}\right)^{2}} \equiv \pm \, q_{0}(r), \end{aligned}$$

11 $$\begin{aligned} y'_{1}(r)=\,&\frac{iy''_{0}(r)}{2y'_{0}(r)}+\frac{iV'(r)}{2(E-V(r))}\nonumber \\&-\frac{1}{2y'_{0}(r)}\left[ \frac{m}{r^2}-\frac{eB}{2}-\frac{V'(r)}{E-V(r)}\left(\frac{m}{r}+\frac{eBr}{2}\right)\right] \nonumber \\ \equiv \,&i\zeta (r)\pm q_{1}(r), \end{aligned}$$

in which

12 $$\begin{aligned} q_{1}(r)=&-\frac{1}{2q_{0}(r)}\left[\frac{m}{r^2}-\frac{eB}{2}-\frac{V'(r)}{E-V(r)}\left(\frac{m}{r}+\frac{eBr}{2}\right)\right]; \end{aligned}$$

13 $$\begin{aligned} \zeta (r)=\,&\frac{y''_{0}(r)}{2y'_{0}(r)}+\frac{V'(r)}{2(E-V(r))}\nonumber \\ =\,&\frac{\mathrm {d}}{\mathrm {d}r}\ln (\sqrt{q_{0}(r)})-\frac{\mathrm {d}}{\mathrm {d}r}\ln (\sqrt{E-V(r)}). \end{aligned}$$

The detailed derivation from Eqs. (4) and (5) to Eq. (13) is given in Part 1 of the supplementary material. Finally, the phase function is obtained by integration, retaining the terms up to order $\hbar$:

14 $$\begin{aligned} y(r)=&\int ^{r}\mathrm {d}r\left[ y'_{0}(r)+\hbar y'_{1}(r)\right] \nonumber \\ =&\int ^{r}\mathrm {d}r\left[ \pm \,  q_{0}(r)+i\hbar \frac{\mathrm {d}}{\mathrm {d}r}\ln \left( \sqrt{q_{0}(r)}\right) \right. \nonumber \\&\left. -i\hbar \frac{\mathrm {d}}{\mathrm {d}r}\ln \left( \sqrt{E-V(r)}\right) \pm \hbar q_{1}(r)\right] \nonumber \\ =&\,i\hbar \ln \left( \sqrt{q_{0}(r)}\right) -i\hbar \ln \left( \sqrt{E-V(r)}\right) \nonumber \\&\pm \int ^{r}\mathrm {d}r\left( q_{0}(r)+\hbar q_{1}(r)\right) . \end{aligned}$$

The radial wave function can then be written as

15 $$\begin{aligned} F(r)=\,&\mathcal {C}\frac{1}{\sqrt{\left( E-V(r)\right) q_{0}(r)}}e^{\pm \frac{i}{\hbar }\int ^{r}\mathrm {d}r\left[ q_{0}(r)+\hbar q_{1}(r)\right] }\nonumber \\&\times \left[ \frac{m}{r}+\frac{eBr}{2}-\hbar \zeta (r)\pm i\left( q_{0}(r)+\hbar q_{1}(r)\right) \right] ; \end{aligned}$$

16 $$\begin{aligned} G(r)=\,&\mathcal {C}\sqrt{\frac{E-V(r)}{q_{0}(r)}}e^{\pm \frac{i}{\hbar }\int ^{r}{\mathrm {d}}r[q_{0}(r)+\hbar q_{1}(r)]}, \end{aligned}$$

where ${\mathcal {C}}$ is the normalization factor.

### Effective potential and quasi-bound states

**Table 1:** Relation between the effective potential $U_{\mathrm {eff}}$ and effective energy $E_{\mathrm {eff}}$, indicating the four situations corresponding to different kinds of wavefunction solutions; notably, quasi-bound states exist in situation (2).

	Situation	Solution of the wavefunction
(1)	$E^2>\big(\frac{m}{R}+\frac{eBR}{2}\big)^2$	Plane wave solutions
(2)	$2eBm<E^2\le \big(\frac{m}{R}+\frac{eBR}{2}\big)^2$	Quasi-bound state solutions
(3)	$2EV_{0}-V_{0}^2+\big(\frac{m}{R}+\frac{eBR}{2}\big)^2<E^2\le 2eBm$	Bound state solutions
(4)	$E^2<2EV_{0}-V_{0}^2+\big(\frac{m}{R}+\frac{eBR}{2}\big)^2$	No solution

**Figure 2:**
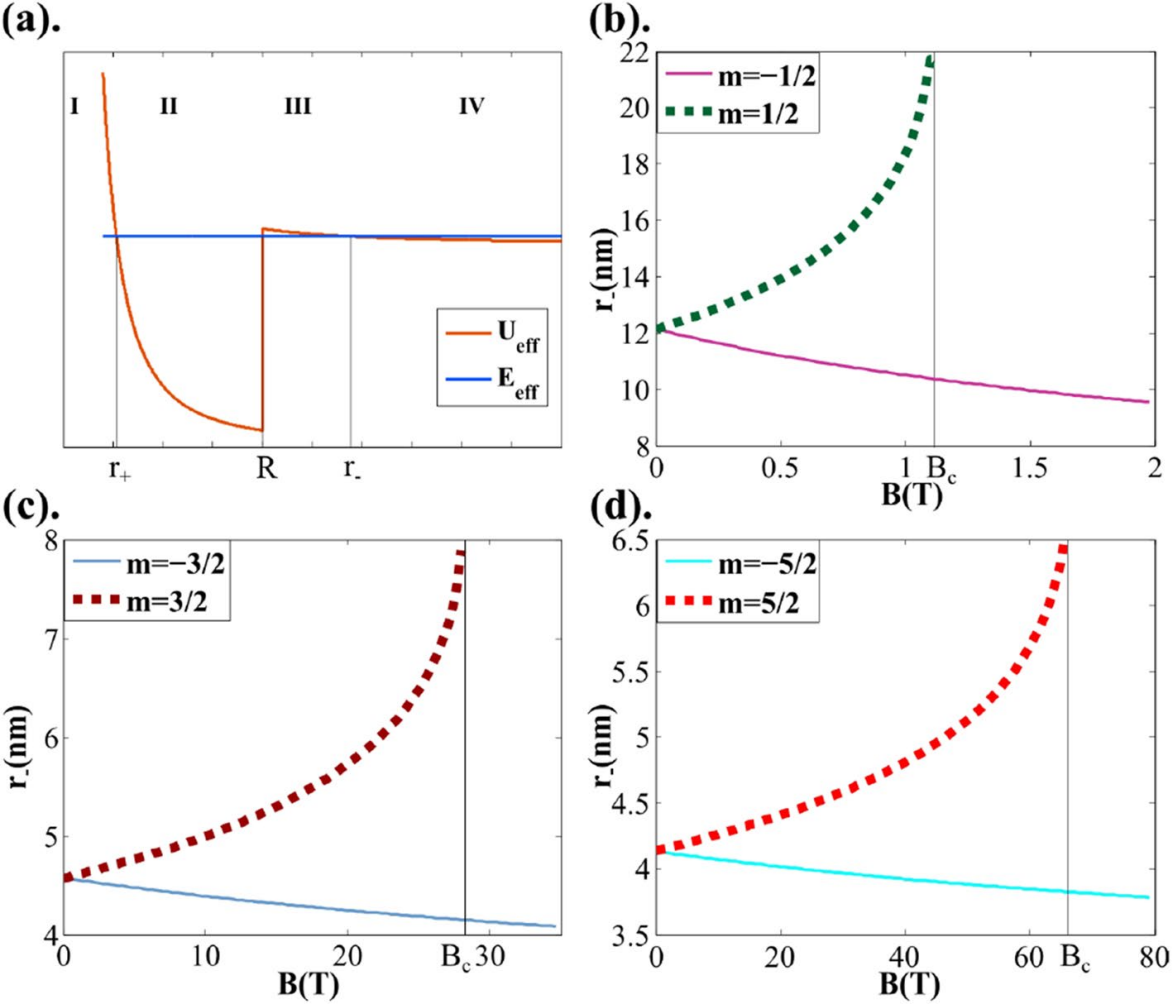
(**a**) Effective energy $E_{eff}$(blue curve) and effective potential $U_{eff}$(red curve) of a graphene quantum dot with radius *R*. The plot can be divided into four regions along the *r* axis (I, II, III, and IV), in which the quasi-bound states exist in region II. Panels (**b**–**d**) plot the $r_{-}$ versus *B* relations for $m=\pm 1/2, \pm 3/2, and \pm 5/2, respectively (R = 4 \mathrm {nm}$). These plots are related to the width of the energy spectrum. The corresponding quasi-bound states become bound states when *B* exceeds the critical magnetic field strength $B_{c}$ ($B_{c}\mid _{m=1/2}\approx 1.1 \mathrm {T}$ for m=1/2; in the higher angular momentum channels, we obtain $B_{c}\mid _{m=3/2}\approx 28 \mathrm {T} and B_{c}\mid _{m=5/2}\approx 66 \mathrm {T}$).

In the following, we set $\hbar =1$ for convenience. To consider the effective momentum, we state $q^2=-U_{\mathrm {eff}}+E_{\mathrm {eff}}$ and $\tilde{q}^2=U_{\mathrm {eff}}-E_{\mathrm {eff}}$, where $E_{\mathrm {eff}}=E^2$ is the effective energy and $U_{\mathrm {eff}}(r,E)=2EV(r)-V^2(r)+(\frac{m}{r}+\frac{eBr}{2})^2$ is the effective potential. Figure 2a shows that the side view of a graphene quantum dot can be divided into four regions along the *r* axis: I (inside), II (inside), III (outside), and IV (outside). Based on the relation between $U_{\mathrm {eff}}$ and $E_{\mathrm {eff}}$, we can also distinguish four situations as shown in Table 1.

This paper focuses only on situation (2), which accommodates quasi-bound states. In situation (2), the turning points $r_{+}$ and $r_{-}$ at which $U_{\mathrm {eff}}=E_{\mathrm {eff}}$ are correspondingly given by

17 $$\begin{aligned} r_{+}=&\left|\frac{|E-V_{0}|-\sqrt{(E-V_{0})^2-2eBm}}{eB}\right|, \end{aligned}$$

18 $$\begin{aligned} r_{-}= \left |\frac{|E|-\sqrt{E^2-2eBm}}{eB} \right|. \end{aligned}$$

Panels (b), (c), and (d) of Fig. 2 plot the dependences of $r_{-}$ on *B* for $m=\pm 1/2, \pm 3/2, and \pm 5/2$, respectively, with $R=4 \mathrm {nm}$.

The wavefunction in the classical allowed region II then follows from Eqs. (15) and (16):

19 $$\begin{aligned} F_{II}(r)=&N e^{\frac{i\pi }{4}}\sqrt{\frac{1}{q_{0}(r)\left( E-V(r)\right) }}\left[ \left( \frac{m}{r}+\frac{eBr}{2}-\zeta (r)\right) \right. \nonumber \\&\times \cos \left( \int _{r_{+}}^{r}\mathrm {d}r\left( q_{0}(r)+q_{1}(r)\right) -\frac{\pi }{4}\right) \nonumber \\&\left. \mp \left( q_{0}(r)+q_{1}(r)\right) \sin \left( \int _{r_{+}}^{r}\mathrm {d}r(q_{0}(r)+q_{1}(r))-\frac{\pi }{4}\right) \right]; \end{aligned}$$

20 $$G_{II}(r)=N e^{\frac{i\pi }{4}}\sqrt{\frac{E-V(r)}{q_{0}(r)}}\cos \left( \int _{r_{+}}^{r}\mathrm {d}r\left( q_{0}(r)+q_{1}(r)\right) -\frac{\pi }{4}\right),$$

where *N* is the normalization factor, and the phase factor $\frac{i\pi }{4}$ is obtained by the Airy function.

In the classical forbidden regions I and III in Fig. 2a, the wavefunction damps exponentially. The radial wavefunction inside the quantum dot can be written as

21 $$\begin{aligned} \psi _{m}^{in}=\left \{ \begin{array}{lcr} \frac{1}{\sqrt{r}} \left( \begin{array}{lcr} \;F_{I}(r)\,\\ \;G_{I}(r)\, \end{array} \right) , r < r_{+}\\ \frac{1}{\sqrt{r}}\left( \begin{array}{lcr} F_{II}(r)\\ G_{II}(r) \end{array} \right) , r_{+} < r < R \end{array}. \right. \end{aligned}$$

The functions $F_{I}(r)$ and $G_{I}(r)$ in Eq. (21) are given in Part 2 of the Supplementary Material. The radially-averaged LDOS is $|\psi _{m}^{in}|^2$.

**Figure 3:**
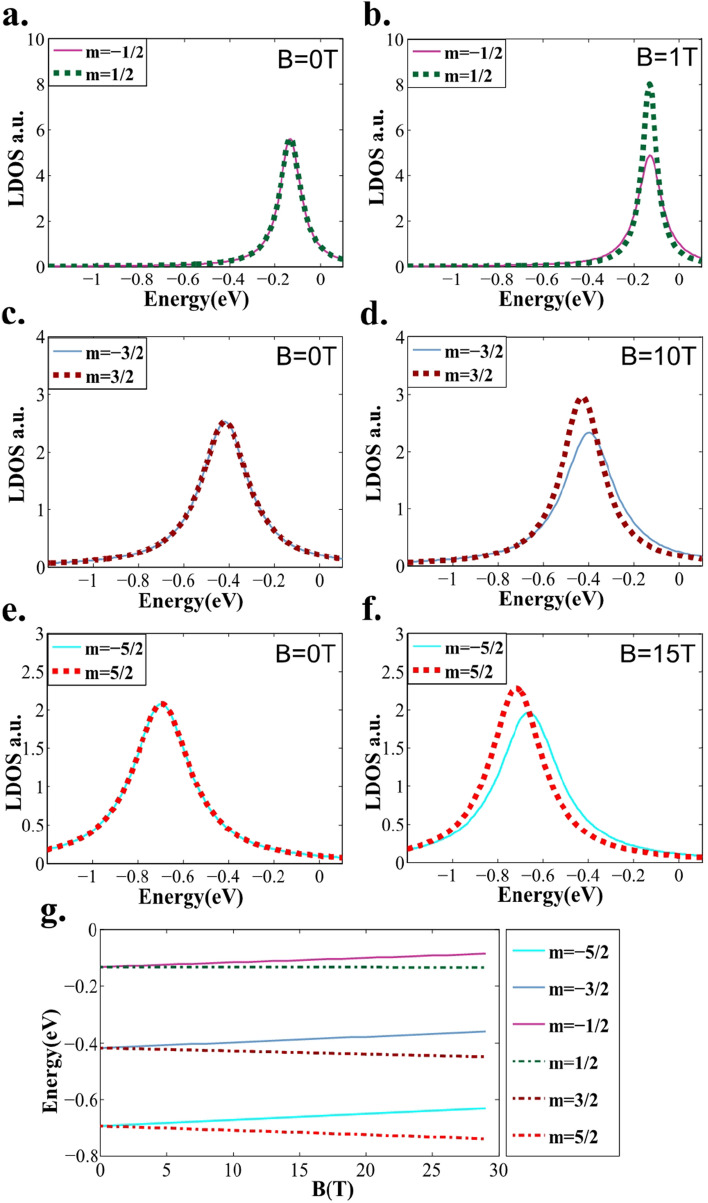
LDOS and energy spectra of quasi-bound states calculated by the WKB approximation. The quasi-bound energy levels of the $m=\pm 1/2,\pm 3/2, and \pm 5/2 \ldots$ states of the graphene quantum dot with $R=4 nm,\,V_0=0.42 eV,\,E_{D}=-0.09 eV$ depend on the magnetic field strength *B*. Panels (a)–(f) show the WKB-approximated LDOS inside the graphene quantum dot under different *B* fields: (**a**) $m=\pm 1/2,B=0$, (**b**) $m=\pm 1/2,B=1 \mathrm {T}$, (**c**) $m=\pm 3/2,B=0$, (**d**) $m=\pm 3/2,B=10 \mathrm {T}$, (**e**) $m=\pm 5/2,B=0$, and (**f**) $m=\pm 5/2,B=15 \mathrm {T}$. Panel (**g**) plots the peak values in the energy spectra as *B* changes from 0 to $30 \mathrm {T},m=\pm 1/2,\pm 3/2, and \pm 5/2$.

At this time, the relation between the angular momentum number *m* and the peak energies of the quasi-bound states can be determined by the quantization rule, explicitly given by

22 $$\begin{aligned}&\int _{r_{+m,n}}^{R}[q_{0m,n}(r)+q_{1m,n}(r)]{\mathrm {d}}r=n\pi +\theta ,n=0,1,2\ldots , \end{aligned}$$

where $q_{0m,n}(r)$ and $q_{1m,n}(r)$ are the functions of the quasi-bound states energy $E_{n,m}$. They are defined by Eqs. (10) and (12), respectively. By applying the quantization rule to Eq. (21), we obtain the relation between $E_{n,m}$ and the quantum number *n*, which can be interpreted as the radial quantum number. $\theta \in (0,\pi )$ is determined by *n*, *m*, and *R* (actually, it largely depends on the shape of $U_{eff}$, which is also related to *m*, *n*, and *R*)^29^.

The width $\Gamma _{m,n}$ of the energy spectrum in the quasi-stationary state is obtained as^27^

23 $$\begin{aligned}&\Gamma _{m,n}=\frac{1}{2\int _{r_{+m,n}}^{R}\frac{E_{n,m}-V(r)}{q_{0m,n}(r)}{\mathrm {d}}r}e^{-2\int _{R}^{r_{-m,n}}[\tilde{q}_{0m,n}(r)+\tilde{q}_{1m,n}(r)]{\mathrm {d}}r}, \end{aligned}$$

where $r_{+m,n}$ and $r_{-m,n}$ are determined by Eqs. (17) and (18), respectively, $E=E_{n,m}$, $\tilde{q}_{0m,n}(r)$, and $\tilde{q}_{1m,n}(r)$ are momenta in the classical forbidden region, as defined by Eq. (S.9) of the supplementary material. The broadening effect mainly originate from the Klein tunneling of Dirac particle. Thus, in our system, the quasi-bound states in the central region have finite life time due to the Klein tunneling, in other words, such quasi-bound state can be called a resonance state.

The above analysis refers to situation (2). When $E^2\le 2eBm$, *B* reaches the critical magnetic field strength $B_{c}=\frac{E^2}{2em}$ (obtained by rearranging Eq. (18)). The situation then transitions from situation (2) to situation (3), in which the quasi-bound states become bound states. Notably, $B_{c}$ and *m* have the same sign; thus, when the magnetic field points upward along the z-direction, $B_{c}$ exists only when $m>0$.

**Figure 4:**
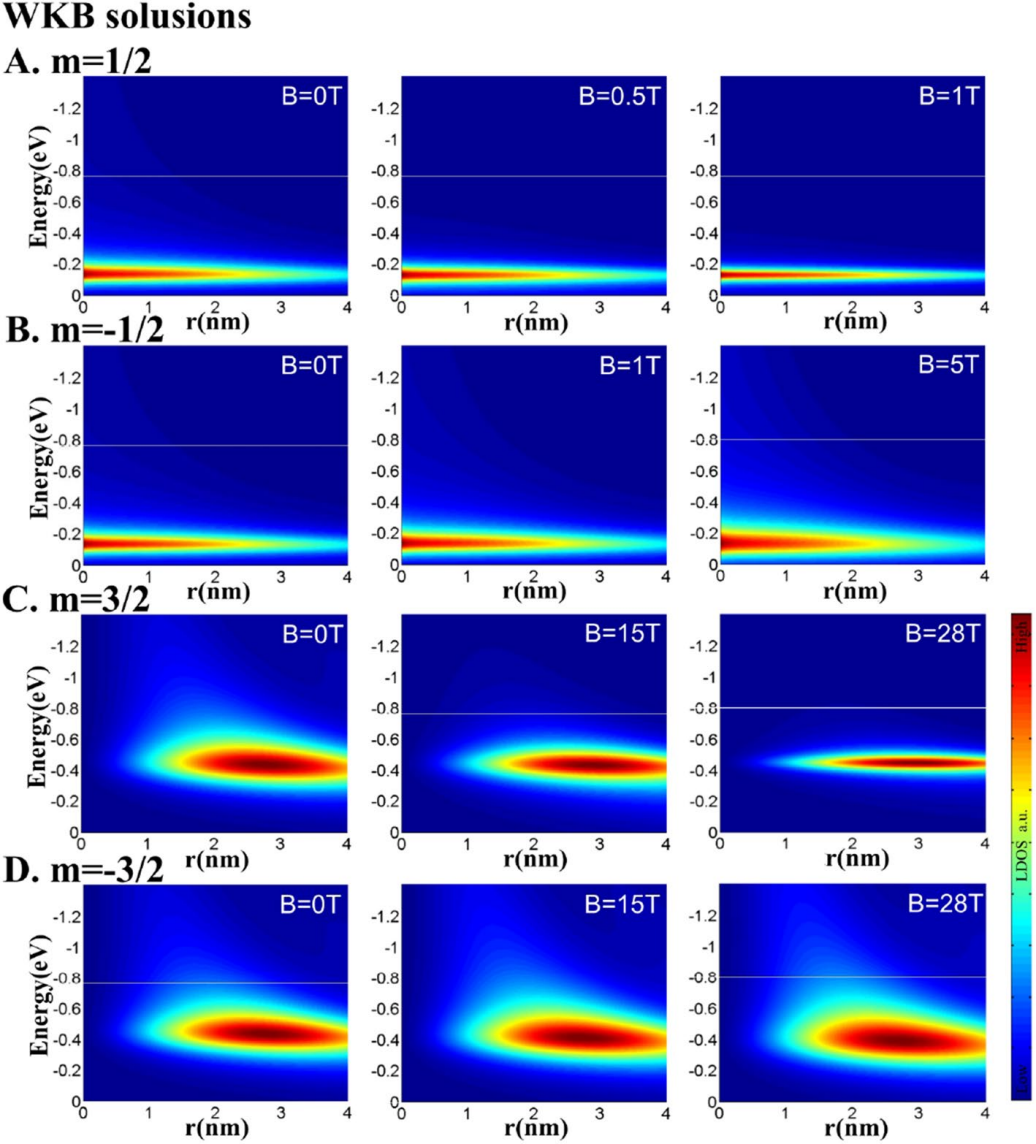
Evolution of LDOS for different angular momenta. Increasing the magnetic field breaks the energy-level degeneracy of the NPN-type graphene quantum dot. Rows (A)–(D) show the three-dimensional LDOS maps of the graphene quantum dot with $$R=4 nm,\,V_0=0.42 eV, and \,E_{D}=-0.09 eV$$ under different magnetic field strengths: (**A**) $$m=1/2,\, B=0,0.5,1 \mathrm {T}\, (B_{c}|_{m=1/2}\approx 1.1 \mathrm {T})$$ , (**B**) $$m=-1/2,\, B=0,1,5 \mathrm {T}\,($$no $$ B_{c}|_{m=-1/2}$$), (**C**) $$m=3/2,\, B=0,15,28 \mathrm {T} \, (B_{c}|_{m=3/2}\approx 28.2 \mathrm {T})$$, and (D) $$m=-3/2,\, B=0,15,28 \mathrm {T}\,($$no $$ B_{c}|_{m=-3/2}) .$$ When $$m>0$$, the energy level shifts in the negative direction and becomes narrower; when $$m>0$$, it moves in the positive direction and widens.

In the following section, we analyze the relation between the energy $E_{0,m}$ of the quasi-bound states and the angular momentum *m* for the case $n=0$.

## Numerical results and discussion

In the absence of a magnetic field, the analytical wavefunction of a quasi-bound state with angular momentum *m* is given by^11^:

24 $$\begin{aligned} \psi _{m}^{in}{\varvec{|}}_{B=0}^{ana}=&\frac{1}{\sqrt{2}}i^{m-\frac{1}{2}}T_{m}e^{im\varphi } \nonumber \\&\cdot \left( \begin{array}{lcr} \;J_{m-\frac{1}{2}}(qr)e^{\frac{-i\varphi }{2}}\,\\ \;i\;\mathrm {sgn}(q)\,J_{m+\frac{1}{2}}(qr)e^{\frac{i\varphi }{2}}\, \end{array}\right)  0<r<R. \end{aligned}$$

The transmissivity is

25 $$\begin{aligned} T_{m}{\varvec{|}}_{B=0}^{ana}=\frac{J_{m-\frac{1}{2}}(kR)H^{(1)}_{m+\frac{1}{2}}(kR) -J_{m+\frac{1}{2}}(kR)H^{(1)}_{m-\frac{1}{2}}(kR)}{J_{m-\frac{1}{2}}(qR)H^{(1)}_{m+\frac{1}{2}}(kR) -\mathrm {sgn}(kq)J_{m+\frac{1}{2}}(qR)H^{(1)}_{m-\frac{1}{2}}(kR)}, \end{aligned}$$

where $q=(E-V_{0})/\hbar ,\;k=E/\hbar \;(v_{F}=1)$, $J_{m\pm \frac{1}{2}}$ is the Bessel function of the first kind, and $H^{(1)}_{m\pm \frac{1}{2}}$ is the Hankel’s function of the first kind. The LDOS inside the graphene quantum dot with no magnetic field is then obtained by noting that $|\psi _{m}^{in}{\varvec{|}}_{B=0,rigorous}|^{2}\varpropto \mathrm {LDOS}(r,E)$.

Notably, for graphene quantum dots without a magnetic field, the spectra of the step-potential and smooth-potential systems share the same features^30^. A comparison of WKB solution and the exact solution when $B=0$ is presented in the Part 3 of the supplementary material. Furthermore, we consider the LDOS and energy spectrum for $B\ne 0$. The solution can be obtained by Eqs. (21) and (22), and the phase shift $\theta$ is determined by comparing the WKB-approximated energy level at $B=0$ with the analytical solution at $B=0$. Panels (a)–(f) of Fig. 3 show the quasi-bound energy levels of $m=\pm 1/2, m=\pm 3/2, and m=\pm 5/2$ as the magnetic field *B* changes from 0 to $30 \mathrm {T}$ upward along the z-axis. The other parameters are $R=4 nm,\,V_0=0.42 eV,\, E_{F}=E+E_{D},\, and E_{D}=-0.09 eV$. Increasing *B* along the z-axis relieved the degeneracy of $\pm m$: for $m>0$, the energy levels $E_{n,m}$ reduced and the bandwidths become increasingly acute until *B* exceeded $B_{c}$; for $m<0$, the energy levels $E_{n,m}$ enlarged and the bandwidths broadened. Fig. 3(g) provides the evolutionary processes of the energy levels as the magnetic field *B* varied. For a given magnetic field strength, increasing the $\mid m\mid$ yielded a more remarkable change in $E_{n,m}$.

Using the results of Fig. 3, Eqs. (21), and (22), we can obtain the transmittance of the graphene quantum dot under a magnetic field. Considering the transmittance as the initial value of the evolution at $r=R$, and applying Eqs. (4) and (5) as the radial evolution equations, we obtained the three-dimensional radial-energy-transmittance LDOS map of the graphene quantum dot under a magnetic field. The results are shown in Fig. 4, which directly reveals the main energy-evolution features in the quasi-bound state of the NPN-type graphene quantum dot under a perpendicular magnetic field: As shown in Fig. 4A and B, the energy degeneracy at $\pm m$ and $B=0$ was relieved at larger *B*. When $m=3/2$, the quasi-bound state energy moved in the negative direction and exhibited sharp resonances at magnetic field strengths above $B_{c}\mid _{m=3/2}\approx 28.2 \mathrm {T}$. Meanwhile, when $m=-3/2$, the energy shift in the quasi-bound state was positive and the energy levels broadened. The evolution of the quasi-bound energy level $E_{m,n}$ depends on the quantization rule given by Eq. (21), and $r_{-}$ is responsible for the energy width $\Gamma _{m,n}$ as shown in Eq. (22). Both of these restriction conditions depend on the magnetic strength *B*. The LDOS at $m=\pm 1/2$ and $m=\pm 3/2$ exhibited similar features, and $B_{c}\mid _{m=1/2} \approx 1.1 \mathrm {T}$ (see Fig. 4 (C), (D)). Moreover, enlarging the |*m*| broadened the energy spectrum by bringing the effective energy closer to the top of the effective potential barrier, thereby increasing the critical magnetic field.

## Conclusion

This paper discussed the evolution of the quasi-bound energy spectrum of an NPN-type graphene quantum dot under a perpendicular magnetic field. The wavefunction was approximately solved using the WKB method. The magnetic field broke the twofold degeneracy of $\pm m$, thereby splitting the energy spectrum and inducing sharp resonances at higher magnetic strengths. The numerical results are relevant to recent experimental results^14,31^. Our method can effectively reveal the evolution of the quasi-bound states in graphene quantum dots placed in magnetic fields.

## Supplementary information

Supplementary Information.
