# Supplementary material for: Quasi-bound states in an NPN-type nanometer-scale graphene quantum dot under a magnetic field
Source: Sci Rep. 2020 Nov 24;10:20426. doi: 10.1038/s41598-020-77357-8 (PMC7686324; doi:10.1038/s41598-020-77357-8)
Supplement: Supplementary file 1 — Supplementary Information. [file 41598_2020_77357_MOESM1_ESM.pdf]

Quasi-bound states in an NPN-type nanometre-scale graphene quantum dot under a magnetic field

Yueting Pan, Haijiao Ji, and Haiwen Liu*

Center for Advanced Quantum Studies, Department of Physics,

Beijing Normal University, Beijing 100875

Xin-Qi Li

Center for Joint Quantum Studies, School of Science,

Tianjin University, Tianjin 300072, China and

Department of Physics, School of Science,

Tianjin University, Tianjin 300072, China

*Electronic address: haiwen.liu@bnu.edu.cn

1. WKB expansion

This part derives Eq. (13) in Section II of the main text from Eqs. (4) and (5). Recall that the simultaneous Eqs. (4) and (5) are respectively given by [1, 2]

$$\hbar\frac{\mathrm{d}F}{\mathrm{d}r}-(\frac{(l+\frac{1}{2})\hbar}{r}+\frac{eBr}{2})F+(E-V(r))G=0;$$
$$\hbar\frac{\mathrm{d}G}{\mathrm{d}r}+(\frac{(l+\frac{1}{2})\hbar}{r}+\frac{eBr}{2})G-(E-V(r))F=0. \tag{S.1}$$

where we set

$$F(r)=\beta(r)e^{i\frac{y(r)}{\hbar}};G(r)=e^{i\frac{y(r)}{\hbar}}. \tag{S.2}$$

Now, inserting Eq. (S.2) into Eq. (S.1), we obtain

$$\beta=\frac{iy'(r)+\frac{m}{r}+\frac{eBr}{2}}{E-V(r)};$$
$$i\hbar y''(r)-y'(r)^2+i\hbar\frac{V'(r)}{E-V(r)}y'(r)$$
$$-(\frac{m}{r}+\frac{eBr}{2})^2+(E-V(r))^2$$
$$-\hbar[\frac{m}{r^2}-\frac{eB}{2}-\frac{(\frac{m}{r}+\frac{eBr}{2})V'(r)}{E-V(r)}]=0. \tag{S.3}$$

The Taylor expansion of y(r) is

$$y(r)=\sum_{n=0}^{\infty}\hbar^n y_n(r). \tag{S.4}$$

Inserting this expression into Eq. (S.3), we obtain

$$i\sum_{n=1}^{\infty}\hbar^n y''_{n-1}(r)-\sum_{n=0}^{\infty}\hbar^n y'_m(r)y'_{n-m}(r)+i\frac{V'(r)}{E-V(r)}\sum_{n-1}^{\infty}\hbar^n y'_{n-1}(r)$$
$$-(\frac{m}{r}+\frac{eBr}{2})^2+(E-V(r))^2$$
$$-\hbar[\frac{m}{r^2}-\frac{eB}{2}-\frac{V'(r)}{E-V(r)}(\frac{m}{r}+\frac{eBr}{2})]=0. \tag{S.5}$$

Equating the coefficients of order $\hbar$, we obtain

$$-y'_0(r)^2-(\frac{m}{r}+\frac{eBr}{2})^2+(E-V(r))^2=0,\ n=0;$$
$$iy''_0(r)-2y'_0(r)y'_1(r)+i\frac{V'(r)}{E-V(r)}y'_0(r)$$
$$-\frac{m}{r^2}+\frac{eB}{2}+\frac{V'(r)}{E-V(r)}(\frac{m}{r}+\frac{eBr}{2})=0,\ n=1. \tag{S.6}$$

The final result

$$
\begin{aligned}
y_0'(r) &= \pm\sqrt{(E-V(r))^2-(\frac{m}{r}+\frac{eBr}{2})^2} \\
&\equiv \pm q_0(r), \\
y_1'(r) &= \frac{iy_0''(r)}{2y_0'(r)}+\frac{iV'(r)}{2(E-V(r))}-\frac{1}{2y_0'(r)}[\frac{m}{r^2}-\frac{eB}{2}-\frac{V'(r)}{E-V(r)}(\frac{m}{r}+\frac{eBr}{2})] \\
&\equiv i\zeta(r)\pm q_1(r),
\end{aligned}
\tag{S.7}
$$

is used in the main text, where

$$
\begin{aligned}
q_1(r) &= -\frac{1}{2q_0(r)}[\frac{m}{r^2}-\frac{eB}{2}-\frac{V'(r)}{E-V(r)}(\frac{m}{r}+\frac{eBr}{2})]; \\
\zeta(r) &= \frac{y_0''(r)}{2y_0'(r)}+\frac{V'(r)}{2(E-V(r))} \\
&= \frac{\mathrm{d}}{\mathrm{d}r}\ln(\sqrt{q_0(r)})-\frac{\mathrm{d}}{\mathrm{d}r}\ln(\sqrt{E-V(r)}).
\end{aligned}
\tag{S.8}
$$

2. Wavefunction in region I

As mentioned in Section III of the main text, the waveform damped exponentially in the classical forbidden region I (see Fig. 2(a)). The effective momenta in the classical forbidden regions are given by[1]:

$$
\begin{aligned}
\tilde{q_0}(r) &= -iq_0(r) = \sqrt{(\frac{m}{r}+\frac{eBr}{2})^2-(E-V(r))^2}; \\
\tilde{y_0'}(r) &= \pm\tilde{q_0}(r); \\
\tilde{q_1}(r) &= -iq_1(r) = \frac{1}{2\tilde{q_0}(r)}[\frac{m}{r^2}-\frac{eB}{2}-\frac{V'(r)}{E-V(r)}(\frac{m}{r}+\frac{eBr}{2})]; \\
\tilde{\zeta}(r) &= \frac{\tilde{y_0''}(r)}{2\tilde{y_0'}(r)}+\frac{V'(r)}{2(E-V(r))} \\
&= \frac{\mathrm{d}}{\mathrm{d}r}\ln(\sqrt{\tilde{q_0}(r)})-\frac{\mathrm{d}}{\mathrm{d}r}\ln(\sqrt{E-V(r)}).
\end{aligned}
\tag{S.9}
$$

Using the Airy function, these results are connected to the wavefunction in region II at the boundaries, and the wavefunction in region I is then obtained as

$$
\begin{aligned}
F_I(r) &= \frac{N}{2}\frac{1}{\sqrt{(E-V(r))\tilde{q_0}(r)}}[\frac{m}{r}+\frac{eBr}{2}-\tilde{\zeta}(r)+\tilde{q_0}(r)+\tilde{q_1}(r)] \\
&\quad\times e^{-\int_r^{r_+}\mathrm{d}r[\tilde{q_0}(r)+\tilde{q_1}(r)]}; \\
G_I(r) &= \frac{N}{2}\sqrt{\frac{E-V(r)}{\tilde{q_0}(r)}}e^{-\int_r^{r_+}\mathrm{d}r[\tilde{q_0}(r)+\tilde{q_1}(r)]}.
\end{aligned}
\tag{S.10}
$$

3. Contrast between the rigorous and WKB solutions

To check the accuracy of our results, we compared our WKB solution with the rigorous solution[3] for $B = 0$. The results are shown in Fig. S.1 below.

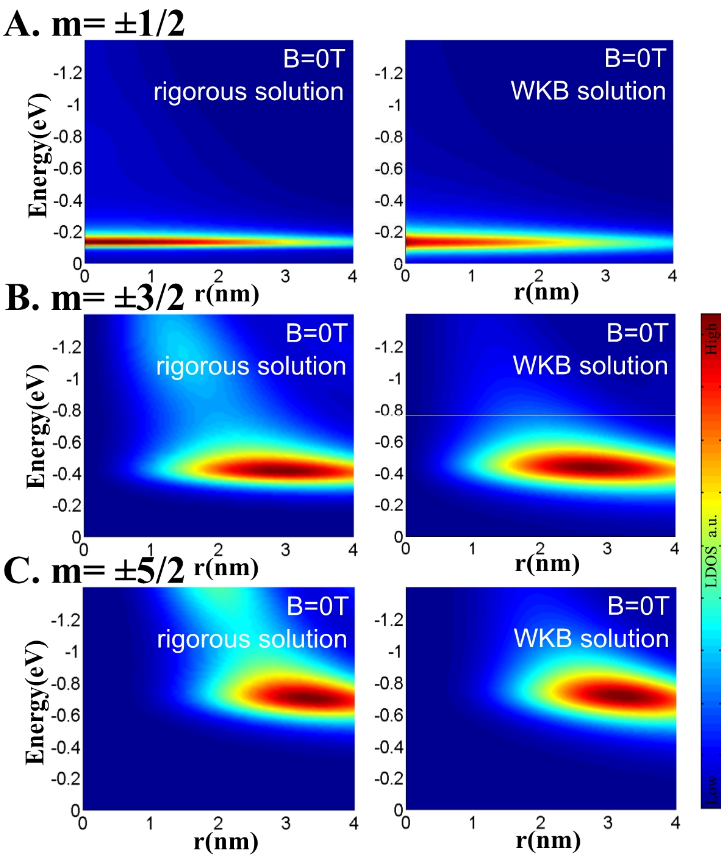

Figure S1: Three-dimensional LDOS maps of the NPN-type graphene quantum dot with $R = 4nm$, $V_0 = 0.42eV$, $E_F = E + E_D$, $E_D = -0.09eV$, $andB = 0$ computed by the rigorous method (left) and WKB method (right): (A) $m = \pm 1/2$, (B) $m = \pm 3/2$, and (C) $m = \pm 5/2$. The energy levels of $\pm m$ are degenerate.

As shown in Fig. S1, the LDOS maps at $B = 0$ obtained by the WKB and rigorous solutions are similar when $m = \pm 1/2, \pm 3/2, \pm 5/2; B = 0$, demonstrating the rationality of the WKB method in our system.

---

[1] JW Van Orden, Sabine Jeschonnek, and John Tjon. Scaling of dirac fermions and the wkb approximation. *Physical Review D*, 72(5):054020, 2005.

[2] VV Rubish, V Yu Lazur, OK Reity, S Chalupka, and M Salak. The wkb method for the dirac equation with the vector and scalar potentials. *Czechoslovak journal of physics*, 54(9):897–919, 2004.

[3] Christopher Gutiérrez, Lola Brown, Cheol-Joo Kim, Jiwoong Park, and Abhay N Pasupathy. Klein tunnelling and electron trapping in nanometre-scale graphene quantum dots. *Nature Physics*, 12(11):1069, 2016.
